# Combined prognostic nutritional index ratio and serum amylase level during the early postoperative period predicts pancreatic fistula following pancreaticoduodenectomy

**DOI:** 10.1186/s12893-020-00838-0

**Published:** 2020-08-06

**Authors:** Teruhisa Sakamoto, Takuki Yagyu, Ei Uchinaka, Masaki Morimoto, Takehiko Hanaki, Joji Watanabe, Manabu Yamamoto, Tomoyuki Matsunaga, Naruo Tokuyasu, Soichiro Honjo, Yoshiyuki Fujiwara

**Affiliations:** Division of Surgical Oncology, Department of Surgery, School of Medicine, Tottori University Faculty of Medicine, 36-1 Nishi-cho, Yonago, 683-8504 Japan

**Keywords:** PNI, Serum amylase level, Pancreatic fistula

## Abstract

**Background:**

The aim of this study was to investigate the usefulness of the range of change in prognostic nutritional index (PNI) during the early postoperative period as a predictor of postoperative pancreatic fistula (POPF) after pancreaticoduodenectomy.

**Methods:**

Data were retrospectively analyzed for 192 patients who underwent pancreaticoduodenectomy. Univariate and multivariate logistic regression analyses were used to evaluate perioperative variables. PNI^P3-Pre^ ratio represented the range of change in PNI from before surgery to postoperative day (POD) 3, PNI^P1-Pre^ ratio represented the range of change in PNI from before surgery to POD 1, and PNI^P3-P1^ ratio represented the range of change in PNI from POD 1 to POD 3.

**Results:**

The area under the curve (AUC) for PNI^P3-P1^ for prediction of POPF following pancreaticoduodenectomy was 0.683 (*P* <  0.001), which was highest among PNI ratios and higher than PNI on POD 3. The AUC for serum amylase level on POD 1 was 0.704 (*P* <  0.001), which was superior to the corresponding AUC on POD 3. The AUC for the combination of PNI^P3-P1^ ratio and serum amylase level on POD 1 for prediction of POPF was higher than the AUC of either indicator alone (0.743, *P* <  0.001). The combination of PNI^P3-P1^ ratio and serum amylase level on POD 1 was an independent predictor of POPF following pancreaticoduodenectomy (*P* = 0.018).

**Conclusions:**

The combination of the range of change in PNI from POD 1 to POD 3 and serum amylase levels on POD 1 may be useful for prediction of POPF following pancreaticoduodenectomy.

## Introduction

Mortality associated with pancreaticoduodenectomy has declined to less than 3% following recent advances in surgical procedures and perioperative managements; however, morbidity remains high [1]. Postoperative pancreatic fistula (POPF), which is the most frequent serious complication following pancreaticoduodenectomy, remains a problem; it may cause life-threatening complications such as intra-abdominal hemorrhage, intra-abdominal abscess, and sepsis, all of which are associated with high mortality rates [2, 3]. Despite extensive efforts to improve surgical outcomes following pancreaticoduodenectomy, no definitive method exists to completely prevent POPF. Therefore, it is necessary to predict POPF accurately during the early postoperative period after pancreaticoduodenectomy, to protect patients from possible severe adverse events.

Risk indicators have been suggested to predict the development of POPF. The prognostic nutritional index (PNI), which is calculated from routine blood tests, is a well-known nutritional indicator, a well-recognized prognostic factor in cancer, and a predictive risk factor for complications in highly-invasive surgeries [4–6]. Several studies have reported a relationship between PNI perioperatively and POPF following pancreaticoduodenectomy [7, 8]. However, to the best of our knowledge, no reports have evaluated the usefulness of the range of change in PNI during the early postoperative period for prediction of POPF following pancreaticoduodenectomy, despite hemodynamic changes in perioperative systemic responses (e.g., inflammation and immunocompetence).

Amylase levels are also a reliable method for prediction of POPF. Drain amylase level has been generally accepted as the main diagnostic indicator of POPF in the International Study Group of Pancreatic Fistula (ISGPF) criteria, and has been used for prediction of POPF worldwide. However, some reports demonstrated that the concentration of drain amylase does not always match the severity of POPF following pancreaticoduodenectomy [9–11]. Several previous studies indicated that an elevated serum amylase level during the early postoperative period was associated with the development of POPF [12–14]. This may reflect the local response from remnant pancreatic tissue, considering that an elevated serum amylase level represents pancreatitis in the remnant pancreatic tissue or local ischemia, which leads to POPF [15, 16]. However, the relationship between PNI (an indicator of the systemic response) and serum amylase level (an indicator of the local response), regarding prediction of POPF after pancreaticoduodenectomy, remains unclear. We speculated that the combination of the range of change in PNI and the serum amylase level, which indicate different patient responses during the early postoperative period, might be superior to using the range of change in PNI alone for prediction of POPF after pancreaticoduodenectomy.

The aim of the present study was to investigate the usefulness of the range of change in PNI during the early postoperative period for prediction of POPF following pancreaticoduodenectomy. An additional aim was to assess the predictive significance of the combination of the range of change in PNI and serum amylase level during the early postoperative period for POPF following pancreaticoduodenectomy.

## Patients and methods

### Patients

Data for 192 consecutive patients who underwent pancreaticoduodenectomy for malignant or benign disease of the pancreatic head and periampullary region at Tottori University Hospital (Yonago, Japan) between January 2008 and July 2019 were retrospectively analyzed.

### Surgical procedures

Standard pancreaticoduodenectomy or pylorus-preserving pancreaticoduodenectomy was performed for the patients enrolled in this study. Reconstruction of the digestive tract was performed using a modified Child’s method in standard pancreaticoduodenectomy or the Traverso method in pylorus-preserving pancreaticoduodenectomy, with a Braun anastomosis. Pancreatojejunostomy was performed with a duct-to-mucosa anastomosis in end-to-side fashion using eight 5–0 absorbable interrupted sutures with either internal or external stents for drainage of pancreatic juice in the main pancreatic duct; for seromuscular–parenchymal anastomosis, either the modified Kakita method or the modified Blumgart method was performed using 3–0 or 4–0 nonabsorbable sutures [17]. After reconstruction of the digestive tract, closed peritoneal drainage tubes were routinely inserted at the superior and inferior sides of the pancreaticojejunostomy and behind the hepaticojejunostomy.

### Postoperative management

Prophylactic antibiotics were routinely administered for 3 days, including the day of surgery, in all patients. Oral intake was usually started on postoperative day (POD) 4 unless postoperative serious complications occurred, such as paralytic ileus or severe pneumonia. No patients received preoperative nutritional support. However, nutritional support (e.g., enteral nutrition via enteral feeding tube or total parenteral nutrition) was administered to patients with poor oral intake after surgery during the perioperative period. Proton pump inhibitors were provided to patients throughout the entire postoperative course.

The amylase concentrations in drain fluids were examined on POD 1 and 3. Concurrent bacterial culture and bacterial smear tests of the drain fluids were performed to detect infection. If the drain amylase levels on POD 3 were less than three-fold greater than serum values or less than < 1000 IU/l and bacterial smear tests of drainage fluid on POD 3 were negative, the drains were removed on POD 3 or 4. In patients with suspected POPF with leakage of the pancreaticojejunostomy or infection, the drains were replaced on POD 7, then once per week. The drains were maintained until POPF was resolved.

POPF was defined in accordance with ISGPF criteria [18]. POPF classified as grade B or C according to ISGPF criteria was considered POPF in this study; patients were divided into a POPF group and a non-POPF group. The upper limit of normal serum amylase in our institution was 132 IU/l.

### Clinicopathological variables

Patients’ medical records were retrospectively reviewed for the following clinicopathological variables: age, sex, body mass index, histological diagnosis, preoperative biliary drainage, pancreatic texture of the remnant pancreas, diameter of the main pancreatic duct, operation time, intraoperative blood loss volume, and the method of pancreaticojejunostomy. Serum amylase and C-reactive protein (CRP) levels were measured on POD 1 and 3. Serum albumin concentration and absolute total lymphocyte count (as components of PNI) were also recorded before surgery and on POD 1 and 3. PNI was calculated as follows: 10 × serum albumin concentration + 0.005 × total lymphocyte count [4].

In the present study, the range of change in PNI during the early postoperative period was defined as the PNI ratio and the values were calculated as follows: PNI^P3-Pre^ ratio, PNI on POD 3 divided by preoperative PNI multiplied by 100; PNI^P1-Pre^ ratio, PNI on POD 1 divided by preoperative PNI multiplied by 100; and PNI^P3-P1^ ratio, PNI on POD 3 divided by PNI on POD 1 multiplied by 100. The PNI^P3-Pre^ ratio represented the range of change in PNI from before surgery to POD 3. PNI^P1-Pre^ indicated the range of change in PNI from before surgery to POD 1, and PNI^P3-P1^ indicated the range of change in PNI from POD 1 to POD 3. The cut off value of drain amylase level on POD 1 was designated as 4000 IU/l, based on the information in a previous report [19].

### Statistical analysis

The results of the analysis of continuous variables were expressed as median with range, and categorical variables were expressed as number (proportion, %). The Chi-square test, Fisher’s exact test, and the Mann–Whitney U test were used to assess comparisons of clinicopathological variables between the two groups; correlations between two variables were evaluated using Spearman’s rank correlation coefficient. Receiver operating characteristic analysis was used to evaluate the predictive significances and areas under the curves (AUCs) for prediction of POPF after pancreaticoduodenectomy. The optimal cutoff values for the PNI ratio, serum amylase level, and serum CRP level for prediction of POPF were also determined using Youden’s index in receiver operating characteristic analysis.

Univariate and multivariate logistic regression analyses were performed to clarify the predictive factors for POPF after receiver operating characteristic analysis. *P* <  0.05 was considered statistically significant, and all statistical analyses were performed using SPSS software (Version 24; IBM Corp., Armonk, NY, USA).

## Results

POPF occurred in 54/192 (28.1%) patients. No patients with removal of drain tubes on POD 3 or 4 required intervention such as drain re-insertion or percutaneous drainage. Postoperative enteral nutrition via enteral feeding tube was performed in 7/192 (3.6%) patients; total parenteral nutrition was performed in 48/192 (25.0%) patients. Nineteen of 192 (9.9%) patients received both enteral nutrition and total parenteral nutrition during the perioperative period.

Table 1 shows the comparison of clinicopathological variables between patients with POPF (POPF group) and patients without POPF (non-POPF group). No significant correlations were observed between the POPF and non-POPF groups in terms of age, operative time, intraoperative blood loss volume, preoperative serum albumin level, preoperative total lymphocyte count, serum CRP level on POD 1, preoperative PNI, PNI on POD 1, or PNI^P1-Pre^ ratio. The proportions of men, presence of preoperative biliary drainage, and soft pancreatic texture of the remnant pancreas were significantly higher in the POPF group than in the non-POPF group. In addition, body mass index was significantly higher in the POPF group than in the non-POPF group. The POPF group had significantly lower proportions of a histological diagnosis of pancreatic ductal adenocarcinoma and shorter diameter of the main pancreatic duct, compared with the non-POPF group. A modified Blumgart anastomosis was performed significantly more frequently in the non-POPF group than in the POPF group. Serum and drain amylase levels on POD 1 and 3 and serum CRP levels on POD 3 were significantly higher in the POPF group than in the non-POPF group. PNI on POD 3, PNI^P3-Pre^ ratio, and PNI^P3-P1^ ratio were significantly lower in the POPF group than in the non-POPF group.

**Table 1:** Patients’ clinicopathological variables

Variables	POPF group (*n* = 54)	non-POPF group (*n* = 138)	*P* value
Age, year, median (range)	69.7 (31–84)	70.9 (17–86)	0.604
Sex, male, (n, %)	42 (77.8%)	79 (57.2%)	0.008
Body mass index, median (range)	23.3 (16.5–29.6)	21.5 (15.1–31.5)	0.001
Histological diagnosis			0.001
pancreatic ductal adenocarcinoma	9 (16.7%)	64 (46.4%)	
acinar sell cancer	0 (0%)	1 (0.7%)	
bile duct cancer	22 (40.7%)	20 (14.5%)	
carcinoma of the papilla of Vater	12 (22.2%)	21 (15.2%)	
IPMN	6 (11.1%)	17 (12.3%)	
PNET	1 (1.9%)	6 (4.3%)	
SPN	1 (1.9%)	1 (0.7%)	
Others	3 (5.6%)	8 (5.8%)	
Preoperative biliary drainage, present, (n, %)	30 (55.6%)	53 (38.4%)	0.031
Pancreatic texture of remnant pancreas, soft, (n, %)	49 (90.7%)	68 (49.3%)	< 0.001
Diameter of main pancreatic duct, median (range), mm	2.6 (1.0–8.9)	4.3 (1.0–12.7)	< 0.001
Operative time, median (range), (min)	545 (403–823)	525 (309–780)	0.271
Intraoperative blood loss, median (range), (ml)	570 (64–4016)	557 (95–2950)	0.240
Method of pancreaticojejunostomy, modified BA, (n, %)	28 (51.9%)	97 (70.3%)	0.016
Preoperative serum albumin level, median (range), (g/dl)	4.0 (1.0–5.2)	4.0 (2.1–4.9)	0.607
Preoperative total lymphocyte count, median (range), (/μl)	1504 (875–3168)	1460 (550–5382)	0.338
Serum amylase level on POD 1, median (range), (IU/l)	432 (136–3953)	246 (12–2324)	< 0.001
Serum amylase level on POD 3, median (range), (IU/l)	102 (28–665)	64 (8–1406)	0.001
Drain amylase level on POD 1, median (range), (IU/l)	8652 (66–985,053)	957.5 (12–65,568)	< 0.001
Drain amylase level on POD 3, median (range), (IU/l)	1313 (450–42,370)	187.5 (4–23,814)	< 0.001
Serum CRP level on POD 1 (range), (mg/dl)	7.83 (4.33–17.76)	7.32 (1.04–20.46)	0.160
Serum CRP level on POD 3 (range), (mg/dl)	21.68 (4.85–37.52)	11.46 (0.88–37.56)	< 0.001
Preoperative PNI, median (range)	48.4 (36.6–59.8)	47.7 (24.6–72.9)	0.439
PNI on POD 1, median (range)	31.3 (22.7–41.6)	31.4 (19.4–46.5)	0.795
PNI on POD 3, median (range)	29.5 (23.5–40.3)	32.4 (22.2–49.1)	0.007
PNI^P1-Pre^ ratio, median (range)	65.3 (49–92)	67.6 (41–108)	0.577
PNI^P3-Pre^ ratio, median (range)	62.4 (46–82)	68.3 (44–111)	0.003
PNI^P3-P1^ ratio, median (range)	94.6 (65–122)	101.7 (75–140)	< 0.001

*PD* pancreaticoduodenectomy, *POPF* postoperative pancreatic fistula, *IPMN* intraductal papillary mucinous neoplasm, *PNET* pancreatic neuroendocrine tumor, *SPN* solid pseudopapillary neoplasm, *BA* Blumgart anastomosis, *POD* postoperative day, *CRP* C-reactive protein, *PNI* prognostic nutritional index

Continuous variables are expressed as median with range

**Fig. 1:**
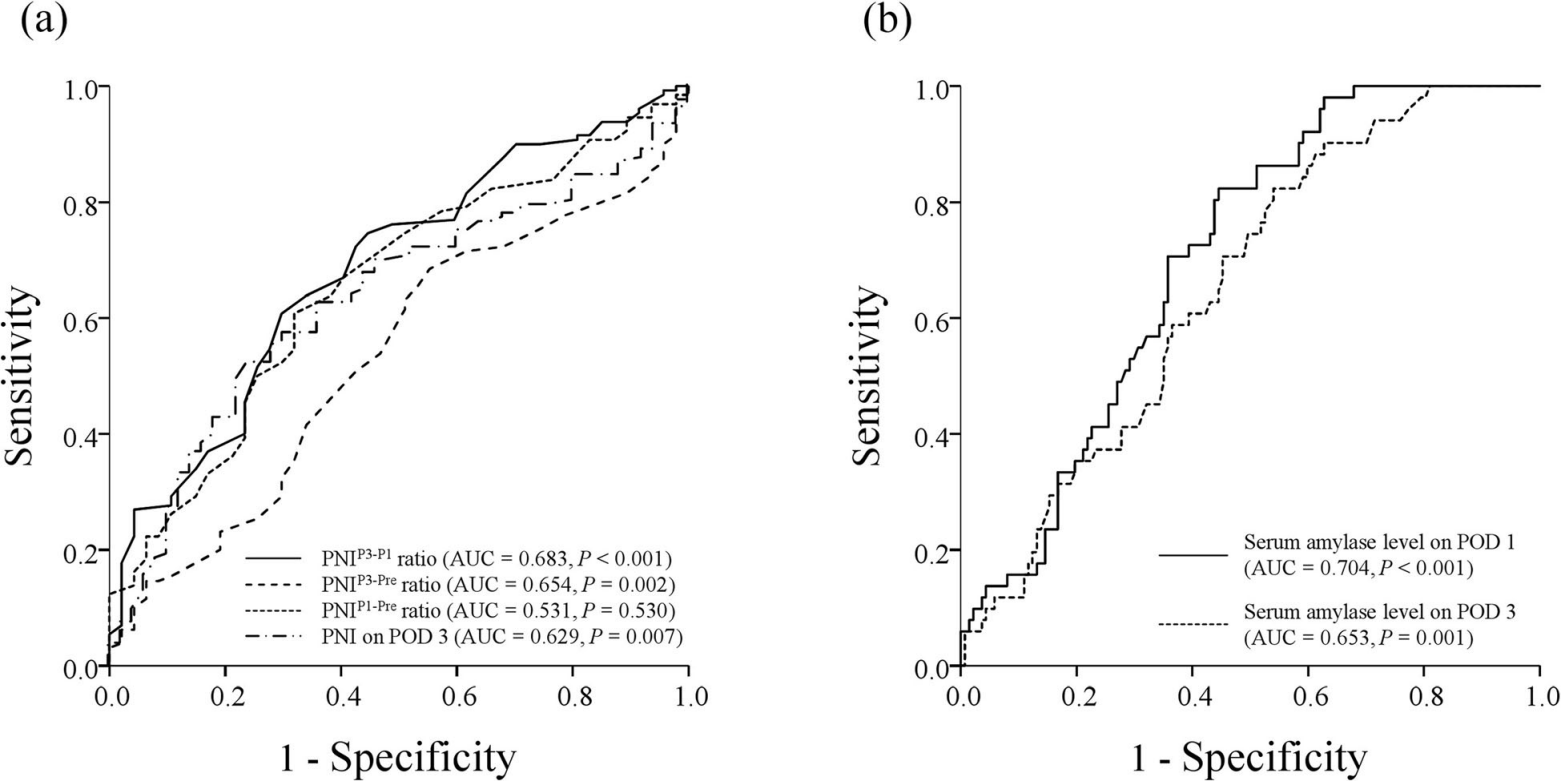
Receiver operating characteristic curves for PNI on POD 3, PNI^P3-P1^ ratio, PNI^P3-Pre^ ratio, and PNI^P1-Pre^ ratio (**a**), and for serum amylase levels on POD 1 and 3 (**b**), for prediction of clinically relevant postoperative pancreatic fistula in patients who underwent pancreaticoduodenectomy. PNI, prognostic nutritional index; AUC, area under the curve; POD, postoperative day

Receiver operating characteristic analysis revealed that the AUC for PNI^P3-P1^ ratio for prediction of POPF following pancreaticoduodenectomy was 0.683 (*P* <  0.001), which was highest among the PNI ratios and higher than PNI on POD 3 (Fig. 1a). Additionally, the AUC for serum amylase level on POD 1 (AUC = 0.704, *P* <  0.001) for prediction of POPF following pancreaticoduodenectomy was higher than the corresponding AUC on POD 3 (Fig. 1b). The optimal cutoff values for PNI^P3-P1^ ratio and serum amylase level on POD 1 for prediction of POPF following pancreaticoduodenectomy were 100 and 268 IU/l, respectively, using the highest Youden indices.

**Fig. 2:**
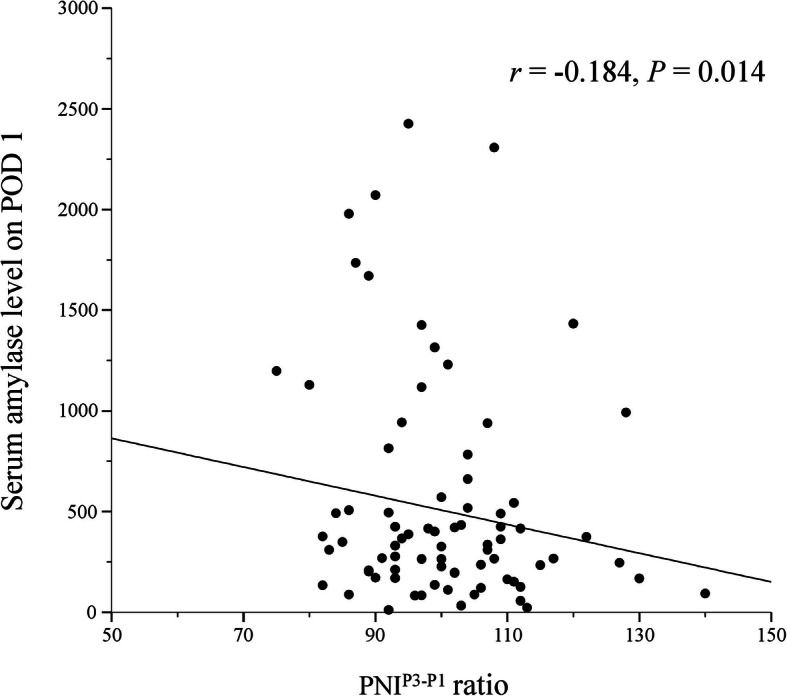
Correlation between PNI^P3-P1^ ratio and serum amylase levels on POD 1. PNI, prognostic nutritional index; POD, postoperative day

**Fig. 3:**
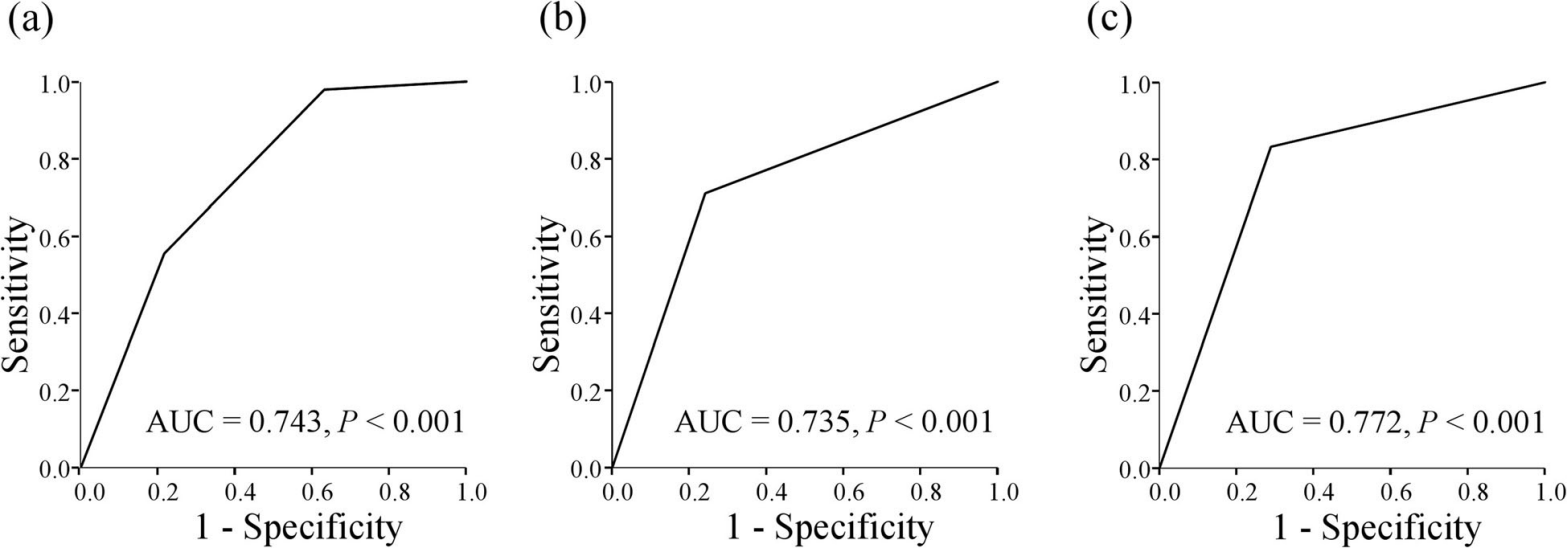
Receiver operating characteristic curves for the combination of PNI^P3-P1^ ratio and serum amylase level on POD 1 (**a**), drain amylase level on POD 1 (**b**), and serum CRP level on POD 3 (**c**) for prediction of clinically relevant postoperative pancreatic fistula in patients who underwent pancreaticoduodenectomy. PNI, prognostic nutritional index; POD, postoperative day; CRP, C-reactive protein; AUC, area under the curve

There was almost no correlation between PNI^P3-P1^ ratio and serum amylase level on POD 1 (*r* = − 0.184, *P* = 0.014; Fig. 2). According to these results, patients were stratified into the following three groups: PNI^P3-P1^ ratio ≥ 100 and serum amylase level on POD 1 < 268, score 2 (*n* = 49); PNI^P3-P1^ ratio ≥ 100 and serum amylase level on POD 1 ≥ 268 or PNI^P3-P1^ ratio < 100 and serum amylase level on POD 1 < 268, score 1 (*n* = 74); and PNI^P3-P1^ ratio < 100 and serum amylase level on POD 1 ≥ 268, score 0 (*n* = 54). The AUC for the combination of PNI^P3-P1^ ratio and serum amylase level on POD 1 for prediction of POPF was 0.743 (*P* <  0.001), indicating that the combination of PNI^P3-P1^ ratio and serum amylase level on POD 1 was more useful than either PNI^P3-P1^ ratio or serum amylase level on POD 1 alone for prediction of POPF following pancreaticoduodenectomy (Fig. 3a). The AUC for drain amylase level on POD 1 for prediction of POPF was 0.735 (*P* <  0.001) (Fig. 3b), which was lower than the AUC for the combination of PNI^P3-P1^ ratio and serum amylase level on POD 1. The AUC for serum CRP level on POD3 was 0.772 (*P* <  0.001, Fig. 3c).

Finally, multivariate analysis revealed that the combination of PNI^P3-P1^ ratio and serum amylase level on POD 1 was an independent predictor of POPF after pancreaticoduodenectomy (*P* = 0.038), as were the presence of preoperative biliary drainage (*P* = 0.019) and serum CRP level on POD3 (*P* <  0.001) (Table 2).

**Table 2:** Univariate and multivariate analyses of the predictive indicators of POPF in patients who underwent pancreaticoduodenectomy

Variables	Univariate analysis			Multivariate analysis		
	Odds ratio	95% CI	*P* value	Odds ratio	95% CI	*P* value
Age (≥ 70 vs. < 70)	0.704	0.374–1.324	0.276			
Sex (male vs. female)	2.614	1266–5.396	0.009	2.514	0.916–6.902	0.074
Body mass index (≥ 25 vs. < 25)	2.738	1.252–5.987	0.012	0.896	0.265–3.025	0.896
Preoperative biliary drainage (present vs. absent)	2.005	1.060–3.791	0.032	3.313	1.216–9.053	0.019
Pancreatic texture of remnant pancreas (soft vs. hard)	10.088	3.791–26.848	< 0.001	2.607	0.533–12.745	0.237
Diameter of main pancreatic duct (≥ 3 mm vs. < 3 mm)	4.070	2.094–7.911	< 0.001	1.396	0.535–3.643	0.495
Operative time (≥ 480 min vs. < 480 min)	1.476	0.719–3.031	0.289			
Intraoperative blood loss (≥ 1000 ml vs. < 1000 ml)	1.663	0.784–3.523	0.185			
Pancreatic stent (internal stent vs. external stent)	0.401	0.182–0.887	0.024	0.378	0.121–1.178	0.094
Drain amylase level on POD 1 (≥ 4000 IU/l vs. < 4000 IU/l)	7.727	3.745–15.941	< 0.001	2.565	0.632–10.417	0.188
Serum CRP level on POD 3 (≥ 17.7 mg/dl vs. < 17.7 mg/dl)	12.226	5.782–25.850	< 0.001	6.929	2.458–19.534	< 0.001
Combination of PNI^P3-P1^ ratio and serum amylase level on POD 1			< 0.001			0.038
score 0 vs. score 2	44.571	5.732–346.559	< 0.001	17.661	1.751–178.140	0.015
score 1 vs. score 2	17.778	2.299–137.497	0.006	9.411	0.961–92.211	0.054

*CI* confidence interval, *BA* Blumgart anastomosis, *POD* postoperative day, *PNI* prognostic nutritional index

## Discussion

In the present study, the results showed that the combination of PNI^P3-P1^ ratio and serum amylase level on POD 1 was a useful indicator for prediction of POPF following pancreaticoduodenectomy. Additionally, our results support the notion that both systemic response-related indicators and local response-related indicators reflect the development of POPF following pancreaticoduodenectomy. POPF following pancreaticoduodenectomy is a serious complication, which leads to poor long-term surgical outcomes, prolonged hospital stay, and increased medical costs. POPF also carries a poor prognosis in malignant disease, secondary to systemic immune function exhaustion or delayed adjuvant therapy [20–22]. Extensive efforts have been made to improve surgical outcomes following pancreaticoduodenectomy; however, there are no definitive methods to prevent POPF. Therefore, although POPF cannot be completely avoided, accurate prediction of POPF early after pancreaticoduodenectomy is needed to prevent secondary life-threatening complications such as intra-abdominal abscess or postpancreatectomy hemorrhage.

PNI as a predictive indicator of POPF was the focus of this study because perioperative nutritional status is an important factor closely associated with postoperative surgical outcomes [23]. PNI is calculated using serum albumin level and peripheral total lymphocyte count; it was originally used to assess perioperative nutritional conditions and postoperative complications in patients with cancer [4]. Albumin is a negative acute-phase marker synthesized by the liver; its levels decrease in the presence of inflammation [24]. Inflammation increases capillary permeability and leakage of serum albumin, leading to expansion of the interstitial space and increasing the distribution volume of albumin. Additionally, the half-life of albumin has been shown to decrease, thereby reducing total albumin mass [25]. Hypoalbuminemia is associated with poor tissue healing, decreased collagen synthesis at anastomoses, and impaired cell-mediated immunity (e.g., macrophage activation and granuloma formation) [26, 27]. Additionally, peripheral total lymphocyte count reflects inflammation and immunity, as well as nutritional status [28–30]; therefore, PNI reflects patients’ systemic response secondary to inflammation, nutritional status, and immunity. Low perioperative PNI reportedly affects the complication rate after pancreaticoduodenectomy, including POPF [7, 8, 31]. Previous studies have indicated the usefulness of PNI perioperatively for prediction of complications after pancreaticoduodenectomy, including POPF. However, the hemodynamics of the inflammatory response occur dynamically; dynamic changes in PNI during the early postoperative period might be more desirable than PNI postoperatively for prediction of POPF following pancreaticoduodenectomy. In fact, previous reports indicated that a steep rise in serum CRP levels from POD 1 to POD 3 was a highly predictive factor for subsequent POPF following pancreaticoduodenectomy [32]. Our results showed that the PNI^P3-P1^ ratio was a useful indicator for prediction of POPF following pancreaticoduodenectomy, reflecting the comprehensive hemodynamic response related to nutrition, inflammation, and immune activity in patients undergoing pancreaticoduodenectomy.

Compared with PNI, serum CRP and albumin are more widely accepted systemic inflammatory markers; in this study, CRP on POD3 was confirmed as an independent predictor of POPF. However, albumin is inferior to PNI for prediction of POPF after pancreaticoduodenectomy because PNI consists of both albumin and lymphocytes. Regarding serum CRP, there are individual differences in the distributions of serum CRP levels related to genetic or environmental factors; there is also a significant association between regulation of serum CRP levels and the presence of polymorphisms in the promoter of interleukin-6 [33]. Therefore, PNI might be more suitable than serum CRP or albumin for prediction of POPF as a factor that changes dynamically during the early postoperative period.

In general, drain amylase level is well-recognized as a predictive factor for POPF; high drain amylase level (≥ 4000 IU/l) on POD1 is reportedly an independent predictive risk factor for the development of POPF [19]. In contrast, several studies indicated the usefulness of postoperative serum amylase level for prediction of POPF following pancreaticoduodenectomy [13, 14, 34–37]. Our results also showed that an elevated serum amylase level on POD 1 was significantly associated with POPF. The mechanism underlying hyperamylasemia in patients with POPF during the early postoperative period after pancreaticoduodenectomy is explained by the finding that postoperative acute pancreatitis, based on elevated serum amylase level on either POD 0 or 1, might be a biochemical manifestation of intraoperative ischemic damage to the pancreatic stump that eventually leads to POPF [38, 39]. For this reason, serum amylase level during the early postoperative period may reflect the local inflammatory response to pancreaticojejunostomy after pancreaticoduodenectomy.

PNI ratio and serum amylase level during the early postoperative period indicate different patient responses after pancreaticoduodenectomy; these two indicators can help to predict POPF following pancreaticoduodenectomy. Almost no correlation between PNI^P3-P1^ ratio and serum amylase level on POD 1 was observed in this study. These results led us to consider that, for prediction of POPF after pancreaticoduodenectomy, the combination of these two indicators would be superior to either measure alone. In fact, the AUC for the combination of PNI^P3-P1^ ratio and serum amylase level on POD 1 was higher than that for either value separately, as well as for drain amylase level on POD1; our results revealed that the combination of PNI^P3-P1^ ratio and serum amylase level on POD 1 was an independent predictive indicator for POPF following pancreaticoduodenectomy.

There were some limitations in our study. First, this was a retrospective analysis, which might have involved bias. Second, we measured PNI before surgery, as well as on POD 1 and 3, and measured serum amylase levels on POD 1 and 3; the optimal times for measurement of these indicators are unclear. Third, PNI after surgery might have been affected by intraoperative administration of exogenous intravenous albumin or blood transfusion in some patients. Fourth, the number of patients included in this study was small; a large-scale, prospective study is needed to verify our results.

## Conclusions

In conclusion, the range of change in PNI from POD 1 to POD 3 was significantly associated with POPF following pancreaticoduodenectomy. Furthermore, the combination of the range of change in PNI from POD 1 to POD 3 and serum amylase levels on POD 1 were useful indicators for prediction of POPF following pancreaticoduodenectomy. These two indicators, inexpensively measured from peripheral blood samples during the early postoperative period, could quickly predict POPF following pancreaticoduodenectomy and might prevent life-threatening complications.

## Data Availability

The datasets used and analyzed during the current study are available from the corresponding author on reasonable request.

## Abbreviations

POPF: Postoperative pancreatic fistula
PNI: Prognostic nutritional index
POD: Postoperative day
CRP: C-reactive protein
AUC: Area under the curve
